# Dendritic Cells in Systemic Lupus Erythematosus: From Pathogenic Players to Therapeutic Tools

**DOI:** 10.1155/2016/5045248

**Published:** 2016-03-30

**Authors:** Jared Klarquist, Zhenyuan Zhou, Nan Shen, Edith M. Janssen

**Affiliations:** ^1^Division of Immunobiology, Cincinnati Children's Hospital Medical Center and the University of Cincinnati College of Medicine, Cincinnati, OH 45229, USA; ^2^Shanghai Institute of Rheumatology, Renji Hospital, School of Medicine, Shanghai Jiao Tong University, Shanghai, China; ^3^Center for Autoimmune Genomics and Etiology (CAGE), Cincinnati Children's Hospital Medical Center, Cincinnati, OH 45229, USA

## Abstract

System lupus erythematosus (SLE) is a multifactorial systemic autoimmune disease with a wide variety of presenting features. SLE is believed to result from dysregulated immune responses, loss of tolerance of CD4 T cells and B cells to ubiquitous self-antigens, and the subsequent production of anti-nuclear and other autoreactive antibodies. Recent research has associated lupus development with changes in the dendritic cell (DC) compartment, including altered DC subset frequency and localization, overactivation of mDCs and pDCs, and functional defects in DCs. Here we discuss the current knowledge on the role of DC dysfunction in SLE pathogenesis, with the focus on DCs as targets for interventional therapies.

## 1. Introduction

Systemic lupus erythematosus is a chronic autoimmune inflammatory disease that affects multiple organ systems, prototypically characterized by high levels of circulating autoantibodies and glomerulonephritis. Clinical symptoms also encompass musculoskeletal, dermatological, neuropsychiatric, pulmonary, gastrointestinal, cardiac, vascular, endocrine, and hematologic manifestations. The reported incidence of SLE nearly tripled over the last 40 years due to improved detection of mild disease [1], but SLE prevalence estimates still vary considerably, ranging from 10 to 150 cases per 100,000, depending on geography, race, and gender [2–5]. In the United States, the prevalence of SLE is higher among Asians, African Americans, African Caribbeans, and Hispanic Americans compared with Caucasians [6–9]. Similarly, in European countries SLE prevalence is higher among people of Asian and African descent [5–9]. Interestingly, SLE is reported infrequently in Africa [10]. Mortality rates are relatively low, at 10–50 per 10,000,000 of the general population and show correlation with renal and cardiovascular manifestations as well as infection [11]. Importantly, patients commonly experience profound fatigue and joint pain and a decreased quality of life [12–15].

The precise etiology of SLE remains unclear and likely varies, considering its diverse clinical manifestations. Nevertheless, SLE is believed to result from dysregulated immune responses, loss of tolerance of CD4 T cells and B cells to ubiquitous self-antigens, and the subsequent production of anti-nuclear and other autoreactive antibodies. This dysregulation is associated with high serum levels of type I IFN, observed in greater than 70% of patients [16, 17]. Current “standard of care” treatments encompass high-dose corticosteroids, antimalarials, and immunosuppressive drugs that are associated with significant adverse side effects. As these treatments suppress symptoms and do not cure the disease, new therapies are needed.

Contemporary treatment strategies have been shifting emphasis toward the identification of immunological processes, both soluble and cellular, in order to redirect aberrant immune responses. Dendritic cells have recently been recognized as important players in the induction and progression of autoimmune diseases, including SLE [18]. Human and mouse studies have associated lupus development with altered DC subset frequency and localization, overactivation of mDCs or pDCs, and functional defects in DCs [19, 20].

However, full dissection of the relative contribution of the causes and the consequences of the dysfunctionality in the different DC subpopulations is needed to understand the processes that govern SLE development, progression, remission, and relapses, in order to design interventional treatments that have the potential to redirect the immune system and eventually lead to a cure for this disease.

## 2. DC Populations in Humans

DCs are a heterogenous population of professional antigen presenting cells, which bridge innate and adaptive immunity. In the absence of exogenous triggers, DCs contribute to the clearance of dying cells and the maintenance of tolerance. During infection, or in the context of autoimmunity, however, DCs play a pivotal role in the activation of CD4 and CD8 T cells. DCs were initially identified by Ralph Steinman and lack typical lineage markers for T cells (CD3), B cells (CD20), and NK cells (CD56) while expressing high levels of MHC class II [35, 36]. Within this population comparative studies have identified a small number of subsets that have homologues in several mammalian species [37, 38].

### 2.1. Myeloid DCs: BDCA1^+^ DCs and BDCA3^+^ DCs

Myeloid DCs are considered “conventional” or “classical” DCs and are characterized by expression of CD11c and CD11b and lack of CD14 and CD16. Within this population we currently distinguish two populations based on the expression of the markers CD1c/BDCA1 and BDCA3/CD141 [39].

The BDCA1^+^ DCs are the major myeloid DC population and are found in blood, lymphoid organs, and most tissues. BDCA1^+^ DCs express a wide variety of pattern recognition receptors including TRL1–8, lectins, and cytokines, allowing them responsiveness to a diverse array of environmental cues. BDCA1^+^ DCs are strong stimulators of naïve CD4 T cell responses, which can be shaped differently depending on which innate stimuli are present [37].

The BDCA3^+^ DCs make up >10% of the mDCs and have been found in lymphoid and nonlymphoid tissues as well as blood and bone marrow. BDCA3^+^ DCs express high levels of TLR3, XCR1, and CLEC9 and have been shown to display an increased capacity to phagocytose dying cells and cross-present cell-associated antigens to CD8 T cells compared to other DCs subsets [34, 40, 41].

### 2.2. Plasmacytoid DCs

pDCs lack the classic mDC markers CD11b and CD11c and express high levels of CD123, CD303 (BDCA2), and CD304 (BDCA4). pDCs are known for their capacity to produce vast amounts of type I IFNs in response to viruses and/or virus-derived nucleic acids predominantly via engagement of TLR7 and TLR9. pDCs have been shown to prime CD4 T cells and cross-prime CD8 T cells, especially in the context of infection [42]. Several studies implicate pDCs in the induction and maintenance of tolerance through the induction of regulatory T cells (Tregs) [43–45].

### 2.3. Monocyte-Associated DCs

There are currently several populations of DCs that are thought to develop from monocytes rather than common DC precursors. These cells display a variety of phenotypes and functions, but there is no consensus on their exact classification or their role* in vivo*.

CD14^+^ DCs are observed in several nonlymphoid tissues, including the skin. These cells express CD11c but lack BDCA1 or BDCA3. The CD14^+^ DCs express low levels of costimulatory molecules or chemokine receptors that promote migration. While these cells have been suggested to be poor at stimulating naïve T cells, they have been found to support the formation of T follicular helper cells and to provide direct help to B cells [46–49].

Inflammatory DCs (iDCs) have been suggested to originate from classic CD14^+^ blood monocytes under inflammatory conditions. These cells may express some of the myeloid DC markers and seem prone to produce proinflammatory cytokines.* In vitro* studies suggest that different types of inflammatory stimuli give rise to populations with distinct proinflammatory phenotypes. TNF*α*/iNOS expressing inflammatory DCs have been found in skin lesions of patients with psoriasis and atopic dermatitis [50, 51].

SlanDCs encompass a subset of monocytes with high expression of MHC class II, CD16, and 6-sulpho LacNAc (slan). SlanDCs were shown to express TRL7 and TLR8 and to produce IL-12, IL-23, and TNF, preferentially promoting Th1 and Th17 cell differentiation. This population has been isolated from the inflamed skin of psoriatic patients and SLE patients with cutaneous lupus, the colon, and draining lymph nodes of patients with inflammatory bowel diseases, as well as CSF samples and inflammatory brain lesions of patients with MS [52–55]. Interestingly, SlanDC infiltration in tumors is associated with tolerance and poor prognosis, indicating either diversity within the slanDC population or heterogeneity in its function.

### 2.4. Tissue DCs

Nonlymphoid tissue resident DCs are present in most tissues in steady state and have been associated initially with induction of tolerance to self-antigens [36–38, 56–58]. These cells migrate at a very low rate to the draining LN under steady state conditions but show significant increased migration under inflammatory conditions. Several studies have identified networks of tissue resident DCs in the skin, lung, gut, and liver [59, 60]. Each of these networks consists of several subpopulations with different capacities for phagocytosis, antigen processing and presentation, migration, and the type of immune response they promote. Due to accessibility, skin DCs, especially Langerhans cells (LC), have been the most studied tissue-DC in the context of SLE.

### 2.5. DC Activation of T Cells

One of the defining features of DCs is the expression of class I and class II major histocompatibility proteins and the processing and presentation of peptide antigens to T cells. DCs predominantly present self-antigens in low quantities resulting in immunologic tolerance. Once activated, however, DCs mature in a process that usually involves migration to a draining lymph node and the priming of T cells [61–63]. The factors governing the functional result of T cell priming are multifactorial, including the relative concentration of surface peptide/MHC, costimulatory molecule expression, and cytokine release. Ultimately, the combination of these signals will result in either T cell anergy, deletion, or activation, proliferation, and differentiation [64–66].

A wide variety of cell surface costimulatory proteins expressed by DCs can signal both activation (41-BB, CD40, CD70, CD80, CD83, CD86, GITRL, ICOSL, LTBR, and OX40L) and inhibition (PDL1, PDL2) of an engaged T cell (reviewed in [67, 68]). In addition, secretion of pro- and anti-inflammatory cytokines by DCs contributes to the outcome of T cell priming. DCs can produce a wide variety of cytokines; which cytokines are produced depends upon environmental signals as well as upon the DC subtype. Cytokine production is driven by input from paracrine and autocrine cytokine signaling, as well as input from innate pattern recognition receptors (PRRs) including toll-like receptors (TLRs). The combination of these signals not only influences whether a T cell becomes activated, but also plays a key role in directing T cell differentiation toward various effector fates.

## 3. Role of DCs in SLE Development and Progression

Although it is not certain how immunological tolerance is broken in SLE, DCs are thought to play key roles [30]. Perhaps the most prominent model proposes that the initial injury is due to a build-up of dying cells, a result of either dysregulated apoptosis or insufficient clearance of dying cells by DCs and other phagocytes [22, 23, 69]. Indeed, high levels of apoptotic cells are found in SLE patient serum, germinal centers, and inflamed tissues, such as the skin and kidney [24, 27]. Mounting evidence indicates that self-RNA and self-DNA from these dying cells induce the unremitting output of type I IFN by pDCs [21] via engagement of TLR9 or TLR7 [31, 70] and potentially via other cytosolic nucleotide sensing pathways such as* RIG-I/IPS1* and STING (*TMEM173*) [28, 71, 72]. Type I IFNs produced by DCs promote their own activation and maturation in an autocrine manner, including increased IFN output and increased surface expression of CD80, CD86, and MHC class II, making them better at activating T cells [21, 25, 26, 73]. Furthermore, type I IFNs directly promote B cell activation, antibody production, and T cell survival and expansion [29, 32, 33]. Altogether, these data suggest that DCs are key players in SLE pathogenesis and point to DCs as promising therapeutic targets.

## 4. DC Abnormalities in SLE Patients

Several reports indicate that the frequency, composition, and phenotype of DCs in SLE patients differ from those of healthy individuals (see Tables 1 and 2). However, it is difficult to compare results between laboratories, given differences in disease activity and manifestations, the effect of various drug treatments on DC development and phenotype, and the variations in analytical parameters.

Studies have shown reduced [74–81], normal [80, 82], and increased [83] levels of CD11c^+^ mDC frequencies in PBMC from lupus patients compared to healthy controls. Similarly, pDC levels were found to be unaffected, reduced [74–78, 84, 85], or increased [79, 86]. Decreased frequencies of pDCs or mDCs were most often associated with active disease and to a lesser degree with nonactive disease [75]. Interestingly, studies showing peripheral pDCs decreases observed a concomitant infiltration of pDCs in nephritic kidneys, suggesting that active pDCs may have migrated to the sites of inflammation [78, 82]. Similarly, Fiore et al. showed that besides pDCs, BDCA1^+^ DCs and BDCA3^+^ DCs were increased in the renal tubulointerstitium of patients with lupus nephritis [78]. Increased numbers of pDCs and inflammatory/slanDCs are also found in cutaneous lesions of lupus patients, further suggesting migration of DCs to target organs [87, 88]. It is likely that DCs that reside in or have been recruited into the affected tissues will display different characteristics than those circulating in the periphery. Consequently, these populations should be included in further assessments in order to understand their contribution to disease pathogenesis and allow for a rational design of DC-targeting therapeutics.

## 5. SLE-Associated Dysfunction in Primary DCs

The few published maturation and functionality studies with primary human DCs have given conflicting results. Earlier reports indicated that DCs from SLE patients have normal or even reduced levels of costimulatory molecules and are poor stimulators of allogeneic T cells in mixed lymphocyte reactions. Scheinecker et al. reported that in SLE patients B7^+^ and CD40^+^ DCs were reduced and that DC-enriched APC from SLE patients displayed a diminished T cell-stimulatory capacity in both the allogeneic and the antigen-specific MLR, as compared with healthy individuals [76]. On the other hand, Mozaffarian et al. showed increased CD80/CD86 and reduced PDL-1 expression on mDC during disease flares and an upregulation of PDL-1 during remission [89]. Similarly, Gerl et al. [81] published that monocytes and mDCs from SLE patients expressed higher levels of CD86 and BAFF, but not CD83 and CD40. Upon further assessment of their migratory capacity, they found that pDCs and mDCs from SLE patients had normal expression of CCR1, CCR5, and CCR7 but reduced expression of the chemokine receptor ChemR23 (CMKLR1). However, pDCs from the SLE patients showed an increased basal and CCL19-specific migration* in vitro*.

Assessment of peripheral monocytes, total DCs, BDCA1^+^ DCs, and CD14^−/low^CD16^+^ DCs by Henriques et al. showed that a higher percentage of SLE monocytes and CD14^−/low^CD16^+^ DCs produced proinflammatory cytokines as well as a higher amount of cytokines produced per cell, particularly in active disease. Data from Kwok et al. [90] seemed to indicate that type I IFN production by pDC upon TLR9 engagement was diminished in SLE patients, leading them to hypothesize that the persistent presence of endogenous IFN*α*-inducing factors induces TLR tolerance in pDCs of SLE patients, resulting in impaired production of IFN*α*. Studies by Jin et al. [79, 91] also suggested deficiencies in TLR9 recruitment/signaling and production of proinflammatory cytokines in pDCs from SLE patients; however, they also showed that SLE pDC had an increased ability to stimulate T cells. Importantly, while pDCs from healthy donors induced suppressive T regulatory cell features (Foxp3 expression) in T cell cultures upon addition of apoptotic PMNs, SLE pDCs failed to do so.

These studies indicate that SLE is associated with phenotypic and functional changes in DCs and that these changes can affect different aspects of the DCs' functional program in distinct and divergent ways.

## 6. SLE-Associated Dysfunction in* In Vitro* Generated DCs

Due to the paucity of DCs in leukopenic SLE patients, many studies have used* in vitro* generated monocyte-derived DCs (moDCs) to gain insight in DC generation, phenotype, and function in the context of SLE.

Initial studies suggest that monocyte-derived DCs had a reduced proinflammatory and T cell stimulatory activity [92] while later studies suggested accelerated differentiation and maturation concomitant with increased activity to maturation stimuli [93]. MoDCs from SLE patients expressed higher levels of HLA-DR and activating Fc*γ*Rs, but decreased expression of inhibitory Fc*γ*R and expression levels correlated with disease severity [92, 94]. In addition, moDCs spontaneously overexpressed activating costimulatory molecules including CD40, CD80, and CD86 and showed increased production of stimulatory cytokines (IL-6, IL-8, and BAFF/BlyS), eventually resulting in an increased capacity to activate T cells in an MLR [93, 95]. Similarly, Nie et al. [96] demonstrated substantial phenotypic and functional aberrations in DCs generated from Flt3-ligand and GM-CSF/IL-4 stimulated bone marrow aspirates. Both immature and mature DCs from SLE donors expressed higher levels of CCR7, CD40, and CD86 and induced stronger T cell proliferation.

## 7. Nature versus Nurture

Drawing causative relationships between DCs frequencies, maturation status, functionality, and disease is complex as it is not clear whether aberrations in DC frequency and functionality are the driver or a result of the disease. It is likely that genetic alterations in DCs predispose to the development of accelerated maturation and abnormal behavior. Evidence for this intrinsic defect is supported by the observations that moDCs from SLE patients, generated from either PBMC or bone marrow, display accelerated maturation and increased proinflammatory status compared to moDC from healthy donors. On the other hand, serum of SLE patients has been shown to contain pro- and anti-inflammatory stimuli like type I IFN, type I IFN-inducing factors, and IL-10 that alter DC differentiation, maturation, and functionality, even in DCs from healthy donors [97–99]. This raises the question whether the aberrant behavior of DCs in SLE patients is a result from an intrinsic defect, a result of their development in an inflammatory environment, or a combination of these two [97]. To further confound the interpretation of human clinical data, various classic SLE treatments, including antimalarials, corticosteroids, and immunosuppressive drugs significantly affect DC number, maturity, and functionality [100].

## 8. Mouse Models to Dissect Role of DCs in SLE Pathogenesis

The availability of mouse models provides an exciting opportunity to gain cellular and molecular insight in the role of different DC populations in the development and progression of SLE. There are a variety of spontaneous models, including the F1 hybrid between the New Zealand Black (NZB) and New Zealand White (NZW) strains (NZB/W F1) and its derivatives, the MRL/lpr and BXSB/Yaa strains, as well as inducible models such as the pristane-induced model and chronic graft-versus-host-disease models (cGVHD) [101–104]. In recent years the number of models has been expanded with genetically modified mice, targeted in genes that can promote, resist, and modify lupus susceptibility [105, 106]. All of these models display their own variation of lupus-like disease reminiscent of symptoms observed in patients, including autoantibody production, lymphoid activation and hyperplasia, lupus nephritis, and skin manifestations. Although all of these models have been instrumental in the identification of several main concepts in this diseases, none of the models can completely recapitulate the complexity and variety of human disease. However, careful pairing of models with patient groups with the similar clinical manifestations can ensure the translational relevance of these preclinical models.

Mouse models have several advantages: (i) the relative homology between human and mouse DCs, (ii) the opportunity to genetically or pharmacologically eliminate specific DC populations during specific stages of disease, (iii) access to all target tissues for the assessment of tissue associated or infiltrating DCs, (iv) the opportunity to assess the effects of common treatments on the parameters, and (v) a plethora of biological and pharmacological tools to dissect the relative contribution of specific molecules and mediators to the development and progression of disease.

## 9. Similarities between Mouse and Human DCs

Recent genomic, proteomic, and functional analyses of mouse and human DCs have identified high homology between the most abundant DC populations [107]. Like in human DCs, mouse DCs lineages encompass conventional DCs, pDCs, CD14^+^ DCs, tissue DCs, and monocyte-derived/inflammatory DCs [38, 108].

Conventional mouse DCs encompass three main subpopulations which are found in circulation as well as in secondary lymphoid organs [109]: (1) CD11c^high^MHCII^+^CD8*α* ^−^33D1^+^Sirp*α* ^+^CD11b^+^ (CD11b DCs), which express most TLRs except Tlr3, display a preference for activation of CD4 T cells, and have high homology with the human BDCA1^+^ DCs; (2) CD11c^high^MHCII^+^CD8*α* ^+^CD205^+^Sirp*α* ^−^CD11b^−^ (CD8*α* DCs), which express Xcl1, CD141, and Clec9A and express mRNAs coding for most TLRs except Tlr5 and Tlr7, and are characterized by high Tlr3 expression; and (3) CD11c^high^MHCII^+^ cells that lack CD8*α*, CD4, and CD11b (generally termed “double” or “triple” negative) DCs that, like CD8a DC, express Xcl1, CD141, Clec9A, and Tlr3 [110–113]. These latter two populations have a high capacity to phagocytose dying cells and cross-present cell-associated or particulate antigens to CD8 T cells. Based on their genomic and functional analysis these two populations are considered to be homologues to the human BDCA3^+^ DCs.

Like human pDCs, mouse pDCs produce vast amounts of type I IFN in response to viruses via TRL7/9 mediated pathways. Compared to their human counterparts, mouse pDCs show relatively poor capacity for phagocytosis and antigen presentation. However, both populations have been implied in the maintenance of peripheral tolerance [45, 114–116].

Various types of inflammatory and monocyte-derived DCs have been identified in mice as well. Tissue infiltrating CD14^+^ DC-like cells have been found under inflammatory conditions [117, 118]. Inflammatory DCs have been shown to arise after a wide variety of immunological insults, including pathogenic infection, experimental sterile inflammation, and models of inflammatory diseases such as RA, colitis experimental autoimmune encephalomyelitis, and allergic asthma (reviewed in [119]).

## 10. The Role of DCs in Mouse SLE Models

Recent studies indicate an important role for DCs in the development and progression of SLE-like disease in mouse models. Similar to human disease, DCs from lupus-prone mice display a range of alterations in their numbers and their functionality [120–123]. Splenic DCs from NZB/W F1 showed enhanced maturation and a stronger ability to attract B cells and present antigens to T cells than DCs from control mice. pDCs from SLE-prone mice showed increased type I IFN producing capacity upon TLR9 stimulation and increased cell survival compared to pDCs from C57BL/6 mice. Enhanced mDC and pDC activity has also been reported in male BXSB/Mp mice that express an extra copy of Tlr7 on the Y chromosome.

Importantly, depletion studies have now shown causal relationships between DC subsets and disease manifestations. Constitutive depletion of pDCs in lupus-prone mice either through genetic ablation of IRF8, a transcription factor required for pDC and CD8*α*DC development, or by diphtheria toxin treatment of mice expressing the diphtheria toxin receptor on pDCs resulted in markedly reduced type I IFN production, a reduced IFN signature, reduced autoantibody production, and reduction in the severity of kidney pathology glomerulonephritis [124–126]. Importantly, transient pDC depletion during the early stages of disease was sufficient to significantly alter the course of the disease, suggesting a more prominent role for pDCs in the induction of the disease than in disease pathogenesis at later stages of disease [125]. Diphtheria toxin treatment of CD11c-DTA MLR.*Fas* ^*lpr*^ mice resulted in reduced T cell differentiation, plasmablast numbers, and autoantibody levels. Interestingly, these mice developed interstitial kidney infiltrates but failed to progress to glomerular or interstitial nephritis, suggesting that DCs play a role in the development of tissue damage [127]. In line with this observation, this group also showed that CD11c depletion, but not LC depletion, resulted in significantly reduced dermatitis, demonstrating that DCs other than LCs control dermatitis in this model [127].

Besides the opportunity to assess the relative and temporal contribution of different DC populations to the development of specific disease manifestations, mouse models also allow for the identification of specific processes in DCs which affect disease development. Targeted deletion of regulatory molecules associated with SLE susceptibility in humans, including Shp1, A20, Blimp-1, Lyn, or Eat-2, specifically in CD11c^+^ cells resulted in increased DC activity and development of inflammatory and autoimmune phenotypes characterized by the production of autoreactive antibodies and several manifestations of SLE, including severe glomerulonephritis [128–132].

Together these observations indicate that mouse models provide a useful platform for the identification, dissection, and targeting of DC intrinsic and extrinsic processes that facilitate the development, progression, and possibly a cure for SLE.

## 11. DC Targeted Therapies for SLE

Based on the general role of DC in the regulation of peripheral tolerance to self-antigens, the dysregulation of DCs observed in SLE, and the emerging evidence of the contribution of DCs in the initiation and perpetuation of SLE pathogenesis, it is not surprising that DC-targeting therapeutic strategies have become a topic of interest. Particularly, strategies that would promote self-antigen presentation in a tolerogenic context could be promising for the generation of an abortive or suppressive environment for the autoreactive T and B cells and restoration of peripheral tolerance [133, 134]. In recent years several* ex vivo* models have been established for the generation of human DCs with stable tolerogenic functions (reviewed in [135]). Generally, these resulting tolerogenic monocyte-derived DCs express low levels of positive costimulatory molecules and high levels of immune suppressive mediators (PDL-1, IL-10, etc.). Upon pulsing with specific antigens these DCs are anticipated to promote antigen-specific tolerance via the induction of T cell anergy, T cell apoptosis, skewing of T cell phenotypes to more Th2 or regulatory phenotypes, and the expansion of regulatory T cells.

Tolerogenic DC therapy is still in its infancy and little data is available on its* in vivo* potential. The first studies showed that transfer of antigen-loaded tolerogenic DCs could induce antigen-specific regulatory CD8 T cells and inhibit effector functions in antigen-specific CD8 T cells [136, 137]. A clinical trial in patients with type I diabetes using DCs treated with antisense oligonucleotides to silence costimulatory molecules was less successful, and although the treatment was well tolerated, only very limited tolerance outcomes were reported [138]. A subsequent trial in T1D patients indicated that transfer of IL-10 and TFG*β*1 generated tolerogenic DCs pulsed with pancreatic islet cells induced antigen-specific T cell hyporesponsiveness and was associated with better glycemic control [139]. Similarly, transfer of a single dose of tolerogenic DCs, derived by* ex vivo* treatment with NF-*κ*B inhibitors, into patients with active RA resulted in a modest improvement in disease activity 3 and 6 months after injection [140]. Currently there are several trials addressing the therapeutic potential of tolerogenic DCs in multiple sclerosis, rheumatoid arthritis, type I diabetes, and allergic asthma [141].

To date no tolerogenic DC transfer studies have been published in preclinical models or SLE patients. However,* in vitro* data indicate that tolerogenic DCs can be generated from SLE patients [83, 142, 143] and that apoptotic cells can be used as source to load the DCs with autoantigens [143]. The insight obtained from currently ongoing tolerogenic DC treatment strategies in other chronic inflammatory diseases will help to identify critical parameters such as dose, route, and duration of treatment leading to the most efficacious outcome [144, 145]. However, a better understanding of the role of DCs in disease pathogenesis is critically needed in order to select the type of tolerogenic DC that can successfully counteract the dysfunctional adaptive immune responses that maintain the disease.

## Figures and Tables

**Table 1:** pDCs in SLE.

Markers used to identify subset	Reference	Frequency	Phenotype	Function
BDCA2^+^ CD123^+^	Tucci et al. [82]	↓ in blood, correlated with LN and ↑ in kidney (more than other DC subsets)		

BDCA2^+^ (blood) and BDCA4^+^ (kidney)	Fiore et al. [78]	↓ in blood in active disease and ↑ in kidney (more than other DC subsets)	DCs in kidney were immature (DC-LAMP^−^), localized to tubulointerstitium, in clusters, and lacked dendrites	

BDCA2^+^ Lin.^−^ HLA-DR^+^	Migita et al. [77]	↓ in blood		

CD123^high^ CD11c^−^ CD16^−^ HLA-DR^+^	Henriques et al. [80]	↓ in blood in active disease		

BDCA2^+^CD123^high^	Kwok et al. [90]	*Normal* in blood	↓ IFN*α* production by PBMC per pDC upon CpG stimulation	

BDCA2^+^ BDCA4^+^ CD123^+^	Jin et al. [79]	↑ in blood per total PBMC	*Normal* HLA-DR, CD86, CD83, CCR7	↑ T cell proliferation in MLR

BDCA2^+^ CD11c^−^	Gerl et al. [81]	na	*Normal* HLA-DR, CD86, CD83, CCR7, CD40, BAFF, CCR1, and CCR5 and ↓ CMKLR1	↑ basal and CCL19-specific migration

BDCA-2^+^ CD4^+^ CD11c^−^ Lin^−^	Hagberg and Rönnblom [86]		↓ SLAMF5/CD84, SLAMF7/CRACC/CD319, *normal* SLAMF1, SLAMF2/CD48, SLAMF3/CD229, SLAMF4/CD244, and SLAMF6/CD352	

**Table 2:** DCs in SLE.

Markers used to identify subset	Reference	Frequency	Phenotype	Function
BDCA1^+^	Fiore et al. [78]	↓ blood in active disease and ↑ kidney in active disease	DCs in kidney were immature (DC-LAMP^−^), localized to tubulointerstitium	

BDCA3^+^	Fiore et al. [78]	↓↓ blood and ↑ kidney in active disease	DCs in kidney were immature (DC-LAMP^−^), localized to tubulointerstitium, with elongated processes	

BDCA1^+^ CD11c^+^ BDCA4^−^ CD19^−^	Jin et al. [91]	↓ in blood per total PBMC	↓ CD83, especially in active disease, *normal* HLA-DR, CD86, and CCR7	

HLA-DR^+^ Lin^−^ CD4^+^	Scheinecker et al. [76]	↓ in blood	↓ CD40^+^, B7^+^, and CD11c^+^	↓ T cell proliferation in MLR

BDCA1^+^ CD11c^+^	Tucci et al. [82]	*Normal* in blood, relatively few in kidney		

CD11c^+^ Lin^−^	Crispín et al. [83]	↑ in blood (though not significant)	↑ CD86^+^, CD80^+^, *normal* HLA-DR^+^, and CD40^+^	*Normal* T cell proliferation in MLR, moDCs fail to increase costimulatory molecule expression upon activation

CD11c^high^ CD14^−^	Gerl et al. [81]	na	↑ CD86, BAFF, *normal* HLA-DR, CD83, CD40, CCR7, CCR1, and CCR5 and ↓ CMKLR1	

Adherent, monocyte-derived DCs (MDDCs)	Ding et al. [93]	na	↑ CD86, CD80, HLA-DR, and CD1a and ↓ CD83 after 5–7 d culture	↑ T cell proliferation in MLR

CD14^+^ sorted, monocyte-derived DCs (MDDCs)	Köller et al. [92]	na	↓ HLA-DR after 8–10 d culture, *normal* CD86, CD83, CD80, CD40, CD54, and CD33	↑ antigen-specific T cell proliferation and normal MLR

M-DC8 (slanDCs)	Hänsel et al. [53]	↑ in skin of patients with cutaneous LE and “strong inflammation” SLE	*In situ* TNF production in cutaneous LE	↑ TNF*α* production by healthy donor slanDCs in response to SLE serum compared with control serum

## References

[1] Uramoto K. M., Michet C. J., Thumboo J., Sunku J., O'Fallon W. M., Gabriel S. E. (1999). Trends in the incidence and mortality of systemic lupus erythematosus, 1950–1992. *Arthritis & Rheumatism*.

[2] Pineles D., Valente A., Warren B., Peterson M., Lehman T., Moorthy L. N. (2011). Worldwide incidence and prevalence of pediatric onset systemic lupus erythematosus. *Lupus*.

[3] Pons-Estel G. J., Alarcón G. S., Scofield L., Reinlib L., Cooper G. S. (2010). Understanding the epidemiology and progression of systemic lupus erythematosus. *Seminars in Arthritis and Rheumatism*.

[4] Lawrence R. C., Helmick C. G., Arnett F. C. (1998). Estimates of the prevalence of arthritis and selected musculoskeletal disorders in the United States. *Arthritis and Rheumatism*.

[5] Danchenko N., Satia J. A., Anthony M. S. (2006). Epidemiology of systemic lupus erythematosus: a comparison of worldwide disease burden. *Lupus*.

[6] Cervera R., Khamashta M. A., Hughes G. R. V. (2009). The Euro-lupus project: epidemiology of systemic lupus erythematosus in Europe. *Lupus*.

[7] Vasudevan A., Krishnamurthy A. N. (2010). Changing worldwide epidemiology of systemic lupus erythematosus. *Rheumatic Disease Clinics of North America*.

[8] Osio-Salido E., Manapat-Reyes H. (2010). Epidemiology of systemic lupus erythematosus in Asia. *Lupus*.

[9] Jakes R. W., Bae S.-C., Louthrenoo W., Mok C.-C., Navarra S. V., Kwon N. (2012). Systematic review of the epidemiology of systemic lupus erythematosus in the Asia-Pacific region: prevalence, incidence, clinical features, and mortality. *Arthritis Care & Research*.

[10] Symmons D. P. (1995). Frequency of lupus in people of African origin. *Lupus*.

[11] Lee Y. H., Choi S. J., Ji J. D., Song G. G. (2016). Overall and cause-specific mortality in systemic lupus erythematosus: an updated meta-analysis. *Lupus*.

[12] Moorthy L. N., Peterson M. G. E., Harrison M. J., Onel K. B., Lehman T. J. A. (2007). Quality of life in children with systemic lupus erythematosus: a review. *Lupus*.

[13] Düzçeker Y., Kanbur N. Ö., Demirkaya E., Derman O., Moorthy L. N., Özen S. (2014). Quality of life measures and psychiatric symptoms in adolescents with systemic lupus erythematosus and familial Mediterranean fever. *International Journal of Adolescent Medicine and Health*.

[14] Waldheim E., Elkan A.-C., Pettersson S. (2013). Health-related quality of life, fatigue and mood in patients with SLE and high levels of pain compared to controls and patients with low levels of pain. *Lupus*.

[15] Yazdany J. (2011). Health-related quality of life measurement in adult systemic lupus erythematosus: Lupus Quality of Life (LupusQoL), Systemic Lupus Erythematosus-Specific Quality of Life Questionnaire (SLEQOL), and Systemic Lupus Erythematosus Quality of Life Questionnaire (L-QoL). *Arthritis Care & Research*.

[16] Baechler E. C., Batliwalla F. M., Karypis G. (2003). Interferon-inducible gene expression signature in peripheral blood cells of patients with severe lupus. *Proceedings of the National Academy of Sciences of the United States of America*.

[17] Bennett L., Palucka A. K., Arce E. (2003). Interferon and granulopoiesis signatures in systemic lupus erythematosus blood. *The Journal of Experimental Medicine*.

[18] Gallo P. M., Gallucci S. (2013). The dendritic cell response to classic, emerging, and homeostatic danger signals. Implications for autoimmunity. *Frontiers in Immunology*.

[19] Ganguly D., Haak S., Sisirak V., Reizis B. (2013). The role of dendritic cells in autoimmunity. *Nature Reviews Immunology*.

[20] Liu J., Cao X. (2015). Regulatory dendritic cells in autoimmunity: a comprehensive review. *Journal of Autoimmunity*.

[21] Elkon K. B., Wiedeman A. (2012). Type I IFN system in the development and manifestations of SLE. *Current Opinion in Rheumatology*.

[22] Elliott M. R., Ravichandran K. S. (2010). Clearance of apoptotic cells: implications in health and disease. *The Journal of Cell Biology*.

[23] Muñoz L. E., Peter C., Herrmann M., Wesselborg S., Lauber K. (2010). Scent of dying cells: the role of attraction signals in the clearance of apoptotic cells and its immunological consequences. *Autoimmunity Reviews*.

[24] Shao W.-H., Cohen P. L. (2011). Disturbances of apoptotic cell clearance in systemic lupus erythematosus. *Arthritis Research & Therapy*.

[25] Gottenberg J.-E., Chiocchia G. (2007). Dendritic cells and interferon-mediated autoimmunity. *Biochimie*.

[26] Baccala R., Hoebe K., Kono D. H., Beutler B., Theofilopoulos A. N. (2007). TLR-dependent and TLR-independent pathways of type I interferon induction in systemic autoimmunity. *Nature Medicine*.

[27] Gaipl U. S., Kuhn A., Sheriff A. (2006). Clearance of apoptotic cells in human SLE. *Current Directions in Autoimmunity*.

[28] Klarquist J., Hennies C. M., Lehn M. A., Reboulet R. A., Feau S., Janssen E. M. (2014). STING-mediated DNA sensing promotes antitumor and autoimmune responses to dying cells. *The Journal of Immunology*.

[29] Hervas-Stubbs S., Perez-Gracia J. L., Rouzaut A., Sanmamed M. F., Le Bon A., Melero I. (2011). Direct effects of type I interferons on cells of the immune system. *Clinical Cancer Research*.

[30] Chan V. S.-F., Nie Y.-J., Shen N., Yan S., Mok M.-Y., Lau C.-S. (2012). Distinct roles of myeloid and plasmacytoid dendritic cells in systemic lupus erythematosus. *Autoimmunity Reviews*.

[31] Celhar T., Magalhães R., Fairhurst A.-M. (2012). TLR7 and TLR9 in SLE: when sensing self goes wrong. *Immunologic Research*.

[32] Hertzog P. J. (2012). Overview. Type I interferons as primers, activators and inhibitors of innate and adaptive immune responses. *Immunology and Cell Biology*.

[33] Marrack P., Kappler J., Mitchell T. (1999). Type I interferons keep activated T cells alive. *The Journal of Experimental Medicine*.

[34] Bachem A., Güttler S., Hartung E. (2010). Superior antigen cross-presentation and XCR1 expression define human CD11c+CD141+ cells as homologues of mouse CD8+ dendritic cells. *Journal of Experimental Medicine*.

[35] Ralph M. S. (2012). Decisions about dendritic cells: past, present, and future. *Immunology*.

[36] O'Keeffe M., Mok W. H., Radford K. J. (2015). Human dendritic cell subsets and function in health and disease. *Cellular and Molecular Life Sciences*.

[37] Collin M., Mcgovern N., Haniffa M. (2013). Human dendritic cell subsets. *Immunology*.

[38] Merad M., Sathe P., Helft J., Miller J., Mortha A. (2013). The dendritic cell lineage: ontogeny and function of dendritic cells and their subsets in the steady state and the inflamed setting. *Annual Review of Immunology*.

[39] Steinman R. M., Idoyaga J. (2010). Features of the dendritic cell lineage. *Immunological Reviews*.

[40] Poulin L. F., Salio M., Griessinger E. (2010). Characterization of human DNGR-1^+^ BDCA3^+^ leukocytes as putative equivalents of mouse CD8*α*
^+^ dendritic cells. *The Journal of Experimental Medicine*.

[41] Jongbloed S. L., Kassianos A. J., McDonald K. J. (2010). Human CD141^+^ (BDCA-3)^+^ dendritic cells (DCs) represent a unique myeloid DC subset that cross-presents necrotic cell antigens. *The Journal of Experimental Medicine*.

[42] Villadangos J. A., Young L. (2008). Antigen-presentation properties of plasmacytoid dendritic cells. *Immunity*.

[43] Swiecki M., Colonna M. (2010). Unraveling the functions of plasmacytoid dendritic cells during viral infections, autoimmunity, and tolerance. *Immunological Reviews*.

[44] Ochando J. C., Homma C., Yang Y. (2006). Alloantigen-presenting plasmacytoid dendritic cells mediate tolerance to vascularized grafts. *Nature Immunology*.

[45] Goubier A., Dubois B., Gheit H. (2008). Plasmacytoid dendritic cells mediate oral tolerance. *Immunity*.

[46] Matthews K., Chung N. P. Y., Klasse P. J., Moore J. P., Sanders R. W. (2012). Potent induction of antibody-secreting B cells by human dermal-derived CD14^+^ dendritic cells triggered by dual TLR ligation. *The Journal of Immunology*.

[47] Penel-Sotirakis K., Simonazzi E., Péguet-Navarro J., Rozières A. (2012). Differential capacity of human skin dendritic cells to polarize CD4^+^T cells into IL-17, IL-21 and IL-22 producing cells. *PLoS ONE*.

[48] de Gruijl T. D., Sombroek C. C., Lougheed S. M. (2006). A postmigrational switch among skin-derived dendritic cells to a macrophage-like phenotype is predetermined by the intracutaneous cytokine balance. *Journal of Immunology*.

[49] Klechevsky E., Morita R., Liu M. (2008). Functional specializations of human epidermal langerhans cells and CD14+ dermal dendritic cells. *Immunity*.

[50] Serbina N. V., Salazar-Mather T. P., Biron C. A., Kuziel W. A., Pamer E. G. (2003). TNF/iNOS-producing dendritic cells mediate innate immune defense against bacterial infection. *Immunity*.

[51] Lowes M. A., Chamian F., Abello M. V. (2005). Increase in TNF-*α* and inducible nitric oxide synthase-expressing dendritic cells in psoriasis and reduction with efalizumab (anti-CD11a). *Proceedings of the National Academy of Sciences of the United States of America*.

[52] Hänsel A., Günther C., Ingwersen J. (2011). Human slan (6-sulfo LacNAc) dendritic cells are inflammatory dermal dendritic cells in psoriasis and drive strong Th17/Th1 T-cell responses. *Journal of Allergy and Clinical Immunology*.

[53] Hänsel A., Günther C., Baran W. (2013). Human 6-sulfo LacNAc (slan) dendritic cells have molecular and functional features of an important pro-inflammatory cell type in lupus erythematosus. *Journal of Autoimmunity*.

[54] Thomas K., Dietze K., Wehner R. (2014). Accumulation and therapeutic modulation of 6-sulfo LacNAc+ dendritic cells in multiple sclerosis. *Neurology: Neuroimmunology & Neuroinflammation*.

[55] Bsat M., Chapuy L., Baba N. (2015). Differential accumulation and function of proinflammatory 6-sulfo LacNAc dendritic cells in lymph node and colon of crohn’s versus ulcerative colitis patients. *Journal of Leukocyte Biology*.

[56] Lech M., Gröbmayr R., Weidenbusch M., Anders H.-J. (2012). Tissues use resident dendritic cells and macrophages to maintain homeostasis and to regain homeostasis upon tissue injury: the immunoregulatory role of changing tissue environments. *Mediators of Inflammation*.

[57] Sillé F. C. M., Visser A., Boes M. (2005). T cell priming by tissue-derived dendritic cells: new insights from recent murine studies. *Cellular Immunology*.

[58] Palucka K., Banchereau J., Mellman I. (2010). Designing vaccines based on biology of human dendritic cell subsets. *Immunity*.

[59] Helft J., Ginhoux F., Bogunovic M., Merad M. (2010). Origin and functional heterogeneity of non-lymphoid tissue dendritic cells in mice. *Immunological Reviews*.

[60] Teunissen M. B. M., Haniffa M., Collin M. P. (2012). Insight into the immunobiology of human skin and functional specialization of skin dendritic cell subsets to innovate intradermal vaccination design. *Current Topics in Microbiology and Immunology*.

[61] Caux C., Ait-Yahia S., Chemin K. (2000). Dendritic cell biology and regulation of dendritic cell trafficking by chemokines. *Springer Seminars in Immunopathology*.

[62] Penna G., Vulcano M., Sozzani S., Adorini L. (2002). Differential migration behavior and chemokine production by myeloid and plasmacytoid dendritic cells. *Human Immunology*.

[63] Randolph G. J., Angeli V., Swartz M. A. (2005). Dendritic-cell trafficking to lymph nodes through lymphatic vessels. *Nature Reviews Immunology*.

[64] Green D. R., Droin N., Pinkoski M. (2003). Activation-induced cell death in T cells. *Immunological Reviews*.

[65] Krammer P. H., Arnold R., Lavrik I. N. (2007). Life and death in peripheral T cells. *Nature Reviews Immunology*.

[66] Schwartz R. H. (2003). T cell anergy. *Annual Review of Immunology*.

[67] Hubo M., Trinschek B., Kryczanowsky F., Tuettenberg A., Steinbrink K., Jonuleit H. (2013). Costimulatory molecules on immunogenic versus tolerogenic human dendritic cells. *Frontiers in Immunology*.

[68] Chen L., Flies D. B. (2013). Molecular mechanisms of T cell co-stimulation and co-inhibition. *Nature Reviews Immunology*.

[69] Munoz L. E., Gaipl U. S., Franz S. (2005). SLE—a disease of clearance deficiency?. *Rheumatology*.

[70] Santiago-Raber M.-L., Baudino L., Izui S. (2009). Emerging roles of TLR7 and TLR9 in murine SLE. *Journal of Autoimmunity*.

[71] Liu Y., Jesus A. A., Marrero B. (2014). Activated STING in a vascular and pulmonary syndrome. *The New England Journal of Medicine*.

[72] Pothlichet J., Niewold T. B., Vitour D., Solhonne B., Crow M. K., Si-Tahar M. (2011). A loss-of-function variant of the antiviral molecule MAVS is associated with a subset of systemic lupus patients. *EMBO Molecular Medicine*.

[73] Crow M. K. (2010). Type I interferon in organ-targeted autoimmune and inflammatory diseases. *Arthritis Research & Therapy*.

[74] Gill M. A., Blanco P., Arce E., Pascual V., Banchereau J., Palucka A. K. (2002). Blood dendritic cells and DC-poietins in systemic lupus erythematosus. *Human Immunology*.

[75] Robak E., Smolewski P., Woźniacka A., Sysa-Jȩdrzejowska A., Robak T. (2004). Clinical significance of circulating dendritic cells in patients with systemic lupus erythematosus. *Mediators of Inflammation*.

[76] Scheinecker C., Zwolfer B., Koller M., Manner G., Smolen J. S. (2001). Alterations of dendritic cells in systemic lupus erythematosus: phenotypic and functional deficiencies. *Arthritis & Rheumatism*.

[77] Migita K., Miyashita T., Maeda Y. (2005). Reduced blood BDCA-2^+^ (lymphoid) and CD11c^+^ (myeloid) dendritic cells in systemic lupus erythematosus. *Clinical and Experimental Immunology*.

[78] Fiore N., Castellano G., Blasi A. (2008). Immature myeloid and plasmacytoid dendritic cells infiltrate renal tubulointerstitium in patients with lupus nephritis. *Molecular Immunology*.

[79] Jin O., Kavikondala S., Sun L. (2008). Systemic lupus erythematosus patients have increased number of circulating plasmacytoid dendritic cells, but decreased myeloid dendritic cells with deficient CD83 expression. *Lupus*.

[80] Henriques A., Inês L., Carvalheiro T. (2012). Functional characterization of peripheral blood dendritic cells and monocytes in systemic lupus erythematosus. *Rheumatology International*.

[81] Gerl V., Lischka A., Panne D. (2010). Blood dendritic cells in systemic lupus erythematosus exhibit altered activation state and chemokine receptor function. *Annals of the Rheumatic Diseases*.

[82] Tucci M., Quatraro C., Lombardi L., Pellegrino C., Dammacco F., Silvestris F. (2008). Glomerular accumulation of plasmacytoid dendritic cells in active lupus nephritis: role of interleukin-18. *Arthritis and Rheumatism*.

[83] Crispín J. C., Vargas-Rojas M. I., Monsiváis-Urenda A., Alcocer-Varela J. (2012). Phenotype and function of dendritic cells of patients with systemic lupus erythematosus. *Clinical Immunology*.

[84] Blomberg S., Eloranta M.-L., Magnusson M., Alm G. V., Rönnblom L. (2003). Expression of the markers BDCA-2 and BDCA-4 and production of interferon-*α* by plasmacytoid dendritic cells in systemic lupus erythematosus. *Arthritis and Rheumatism*.

[85] Vallin H., Blomberg S., Alm G. V., Cederblad B., Rönnblom L. (1999). Patients with systemic lupus erythematosus (SLE) have a circulating inducer of interferon-alpha (IFN-*α*) production acting on leucocytes resembling immature dendritic cells. *Clinical and Experimental Immunology*.

[86] Hagberg N., Rönnblom L. (2015). Systemic lupus erythematosus—a disease with a dysregulated type I interferon system. *Scandinavian Journal of Immunology*.

[87] Blomberg S., Eloranta M. L., Cederblad B., Nordlind K., Alm G. V., Rönnblom L. (2001). Presence of cutaneous interferon-*α* producing cells in patients with systemic lupus erythematosus. *Lupus*.

[88] Farkas L., Beiske K., Lund-Johansen F., Brandtzaeg P., Jahnsen F. L. (2001). Plasmacytoid dendritic cells (natural interferon-*α*/*β*-producing cells) accumulate in cutaneous lupus erythematosus lesions. *The American Journal of Pathology*.

[89] Mozaffarian N., Wiedeman A. E., Stevens A. M. (2008). Active systemic lupus erythematosus is associated with failure of antigen-presenting cells to express programmed death ligand-1. *Rheumatology*.

[90] Kwok S.-K., Lee J.-Y., Park S.-H. (2008). Dysfunctional interferon-*α* production by peripheral plasmacytoid dendritic cells upon Toll-like receptor-9 stimulation in patients with systemic lupus erythematosus. *Arthritis Research and Therapy*.

[91] Jin O., Kavikondala S., Mok M.-Y. (2010). Abnormalities in circulating plasmacytoid dendritic cells in patients with systemic lupus erythematosus. *Arthritis Research & Therapy*.

[92] Köller M., Zwölfer B., Steiner G., Smolen J. S., Scheinecker C. (2004). Phenotypic and functional deficiencies of monocyte-derived dendritic cells in systemic lupus erythematosus (SLE) patients. *International Immunology*.

[93] Ding D., Mehta H., McCune W. J., Kaplan M. J. (2006). Aberrant phenotype and function of myeloid dendritic cells in systemic lupus erythematosus. *The Journal of Immunology*.

[94] Carreño L. J., Pacheco R., Gutierrez M. A., Jacobelli S., Kalergis A. M. (2009). Disease activity in systemic lupus erythematosus is associated with an altered expression of low-affinity Fc*γ* receptors and costimulatory molecules on dendritic cells. *Immunology*.

[95] Decker P., Kötter I., Klein R., Berner B., Rammensee H.-G. (2006). Monocyte-derived dendritic cells over-express CD86 in patients with systemic lupus erythematosus. *Rheumatology*.

[96] Nie Y. J., Mok M. Y., Chan G. C. (2010). Phenotypic and functional abnormalities of bone marrow-derived dendritic cells in systemic lupus erythematosus. *Arthritis Research & Therapy*.

[97] Blanco P., Palucka A. K., Gill M., Pascual V., Banchereau J. (2001). Induction of dendritic cell differentiation by IFN-*α* in systemic lupus erythematosus. *Science*.

[98] Decker P., Singh-Jasuja H., Haager S., Kötter I., Rammensee H.-G. (2005). Nucleosome, the main autoantigen in systemic lupus erythematosus, induces direct dendritic cell activation via a MyD88-independent pathway: consequences on inflammation. *Journal of Immunology*.

[99] Sun Z., Zhang R., Wang H. (2012). Serum IL-10 from systemic lupus erythematosus patients suppresses the differentiation and function of monocyte-derived dendritic cells. *Journal of Biomedical Research*.

[100] Shodell M., Shah K., Siegal F. P. (2003). Circulating human plasmacytoid dendritic cells are highly sensitive to corticosteroid administration. *Lupus*.

[101] McGaha T. L., Madaio M. P. (2014). Lupus nephritis: animal modeling of a complex disease syndrome pathology. *Drug Discovery Today: Disease Models*.

[102] Crampton S. P., Morawski P. A., Bolland S. (2014). Linking susceptibility genes and pathogenesis mechanisms using mouse models of systemic lupus erythematosus. *Disease Models & Mechanisms*.

[103] Liu Z., Davidson A. (2013). IFNalpha inducible models of murine SLE. *Frontiers in Immunology*.

[104] Furukawa F., Yoshimasu T. (2005). Animal models of spontaneous and drug-induced cutaneous lupus erythematosus. *Autoimmunity Reviews*.

[105] Perry D., Sang A., Yin Y., Zheng Y.-Y., Morel L. (2011). Murine models of systemic lupus erythematosus. *Journal of Biomedicine and Biotechnology*.

[106] Sang A., Yin Y., Zheng Y.-Y., Morel L. (2012). Animal models of molecular pathology: systemic lupus erythematosus. *Progress in Molecular Biology and Translational Science*.

[107] Crozat K., Guiton R., Guilliams M. (2010). Comparative genomics as a tool to reveal functional equivalences between human and mouse dendritic cell subsets. *Immunological Reviews*.

[108] Miloud T., Hammerling G. J., Garbi N. (2010). Review of murine dendritic cells: types, location, and development. *Dendritic Cell Protocols*.

[109] Thacker R. I., Janssen E. M. (2012). Cross-presentation of cell-associated antigens by mouse splenic dendritic cell populations. *Frontiers in Immunology*.

[110] Reboulet R. A., Hennies C. M., Garcia Z., Nierkens S., Janssen E. M. (2010). Prolonged antigen storage endows merocytic dendritic cells with enhanced capacity to prime anti-tumor responses in tumor-bearing mice. *The Journal of Immunology*.

[111] Katz J. D., Ondr J. K., Opoka R. J., Garcia Z., Janssen E. M. (2010). Merocytic dendritic cells break T cell tolerance to *β* cell antigens in nonobese diabetic mouse diabetes. *Journal of Immunology*.

[112] Shortman K., Heath W. R. (2010). The CD8^+^ dendritic cell subset. *Immunological Reviews*.

[113] Bedoui S., Prato S., Mintern J. (2009). Characterization of an immediate splenic precursor of CD8^+^ dendritic cells capable of inducing antiviral T cell responses. *Journal of Immunology*.

[114] Kool M., van Nimwegen M., Willart M. A. M. (2009). An anti-inflammatory role for plasmacytoid dendritic cells in allergic airway inflammation. *The Journal of Immunology*.

[115] Daissormont I. T. M. N., Christ A., Temmerman L. (2011). Plasmacytoid dendritic cells protect against atherosclerosis by tuning T-cell proliferation and activity. *Circulation Research*.

[116] Ito T., Yang M., Wang Y.-H. (2007). Plasmacytoid dendritic cells prime IL-10-producing T regulatory cells by inducible costimulator ligand. *The Journal of Experimental Medicine*.

[117] Langlet C., Tamoutounour S., Henri S. (2012). CD64 expression distinguishes monocyte-derived and conventional dendritic cells and reveals their distinct role during intramuscular immunization. *Journal of Immunology*.

[118] Plantinga M., Guilliams M., Vanheerswynghels M. (2013). Conventional and monocyte-derived CD11b^+^ dendritic cells initiate and maintain T helper 2 cell-mediated immunity to house dust mite allergen. *Immunity*.

[119] Segura E., Amigorena S. (2013). Inflammatory dendritic cells in mice and humans. *Trends in Immunology*.

[120] Zhu J., Liu X., Xie C. (2005). T cell hyperactivity in lupus as a consequence of hyperstimulatory antigen-presenting cells. *The Journal of Clinical Investigation*.

[121] Hanada T., Yoshida H., Kato S. (2003). Suppressor of cytokine signaling-1 is essential for suppressing dendritic cell activation and systemic autoimmunity. *Immunity*.

[122] Colonna L., Dinnal J.-A., Shivers D. K., Frisoni L., Caricchio R., Gallucci S. (2006). Abnormal costimulatory phenotype and function of dendritic cells before and after the onset of severe murine lupus. *Arthritis Research and Therapy*.

[123] Wan S., Xia C., Morel L. (2007). IL-6 produced by dendritic cells from lupus-prone mice inhibits CD4+CD25+ T cell regulatory functions. *Journal of Immunology*.

[124] Davison L. M., Jørgensen T. N. (2015). Sialic acid-binding immunoglobulin-type lectin H-positive plasmacytoid dendritic cells drive spontaneous lupus-like disease development in B6.Nba2 mice. *Arthritis and Rheumatology*.

[125] Rowland S. L., Riggs J. M., Gilfillan S. (2014). Early, transient depletion of plasmacytoid dendritic cells ameliorates autoimmunity in a lupus model. *The Journal of Experimental Medicine*.

[126] Baccala R., Gonzalez-Quintial R., Blasius A. L. (2013). Essential requirement for IRF8 and SLC15A4 implicates plasmacytoid dendritic cells in the pathogenesis of lupus. *Proceedings of the National Academy of Sciences of the United States of America*.

[127] Teichmann L. L., Ols M. L., Kashgarian M., Reizis B., Kaplan D. H., Shlomchik M. J. (2010). Dendritic cells in lupus are not required for activation of T and B cells but promote their expansion, resulting in tissue damage. *Immunity*.

[128] Talaei N., Yu T., Manion K., Bremner R., Wither J. E. (2015). Identification of the SLAM adapter molecule EAT-2 as a lupus-susceptibility gene that acts through impaired negative regulation of dendritic cell signaling. *The Journal of Immunology*.

[129] Kim S. J., Zou Y. R., Goldstein J., Reizis B., Diamond B. (2011). Tolerogenic function of Blimp-1 in dendritic cells. *Journal of Experimental Medicine*.

[130] Kool M., van Loo G., Waelput W. (2011). The ubiquitin-editing protein A20 prevents dendritic cell activation, recognition of apoptotic cells, and systemic autoimmunity. *Immunity*.

[131] Kaneko T., Saito Y., Kotani T. (2012). Dendritic cell-specific ablation of the protein tyrosine phosphatase Shp1 promotes Th1 cell differentiation and induces autoimmunity. *Journal of Immunology*.

[132] Lamagna C., Scapini P., van Ziffle J. A., DeFranco A. L., Lowell C. A. (2013). Hyperactivated MyD88 signaling in dendritic cells, through specific deletion of Lyn kinase, causes severe autoimmunity and inflammation. *Proceedings of the National Academy of Sciences of the United States of America*.

[133] Schmidt S. V., Nino-Castro A. C., Schultze J. L. (2012). Regulatory dendritic cells: there is more than just immune activation. *Frontiers in Immunology*.

[134] Thomson A. W., Robbins P. D. (2008). Tolerogenic dendritic cells for autoimmune disease and transplantation. *Annals of the Rheumatic Diseases*.

[135] Gordon J. R., Ma Y., Churchman L., Gordon S. A., Dawicki W. (2014). Regulatory dendritic cells for immunotherapy in immunologic diseases. *Frontiers in Immunology*.

[136] Dhodapkar M. V., Steinman R. M., Krasovsky J., Munz C., Bhardwaj N. (2001). Antigen-specific inhibition of effector T cell function in humans after injection of immature dendritic cells. *Journal of Experimental Medicine*.

[137] Dhodapkar M. V., Steinman R. M. (2002). Antigen-bearing immature dendritic cells induce peptide-specific CD8+ regulatory T cells in vivo in humans. *Blood*.

[138] Giannoukakis N., Phillips B., Finegold D., Harnaha J., Trucco M. (2011). Phase I (safety) study of autologous tolerogenic dendritic cells in type 1 diabetic patients. *Diabetes Care*.

[139] Segovia-Gamboa N., Rodríguez-Arellano M. E., Rangel-Cruz R. (2014). Tolerogenic dendritic cells induce antigen-specific hyporesponsiveness in insulin- and glutamic acid decarboxylase 65-autoreactive T lymphocytes from type 1 diabetic patients. *Clinical Immunology*.

[140] Thomas R., Street S., Ramnoruth N. (2011). Safety and preliminary evidence of efficacy in a phase I clinical trial of autologous tolerising dendritic cells exposed to citrullinated peptides (Rheumavax) in patients with rheumatoid arthritis. *Annals of the Rheumatic Diseases*.

[141] Inaba K., Steinman R. M., Pack M. W. (1992). Identification of proliferating dendritic cell precursors in mouse blood. *Journal of Experimental Medicine*.

[142] Wu H. J., Lo Y., Luk D., Lau C. S., Lu L., Mok M. Y. (2015). Alternatively activated dendritic cells derived from systemic lupus erythematosus patients have tolerogenic phenotype and function. *Clinical Immunology*.

[143] Torres Baeza A., Vega Tapia F., Mackern-Oberti J. Generation of tolerogenic dendritic cells from systemic lupus erythematosus patients. http://www.frontiersin.org/10.3389/conf.fimmu.2015.05.00095/event_abstract.

[144] Llanos C., Mackern-Oberti J. P., Vega F., Jacobelli S. H., Kalergis A. M. (2013). Tolerogenic dendritic cells as a therapy for treating lupus. *Clinical Immunology*.

[145] Mok M. Y. (2015). Tolerogenic dendritic cells: role and therapeutic implications in systemic lupus erythematosus. *International Journal of Rheumatic Diseases*.

